# Signaling *via* Class IA Phosphoinositide 3-Kinases (PI3K) in Human, Breast-Derived Cell Lines

**DOI:** 10.1371/journal.pone.0075045

**Published:** 2013-10-04

**Authors:** Veronique Juvin, Mouhannad Malek, Karen E. Anderson, Carine Dion, Tamara Chessa, Charlotte Lecureuil, G. John Ferguson, Sabina Cosulich, Phillip T. Hawkins, Len R. Stephens

**Affiliations:** 1 The Babraham Institute, Babraham, Cambridge, United Kingdom; 2 Astrazeneca, Macclesfield United Kingdom; II Università di Napoli, Italy

## Abstract

We have addressed the differential roles of class I Phosphoinositide 3-kinases (PI3K) in human breast-derived MCF10a (and iso-genetic derivatives) and MDA-MB 231 and 468 cells. Class I PI3Ks are heterodimers of p110 catalytic (α, β, δ and γ) and p50–101 regulatory subunits and make the signaling lipid, phosphatidylinositol (3,4,5)-trisphosphate (PtdIns(3,4,5)P_3_) that can activate effectors, eg protein kinase B (PKB), and responses, eg migration. The PtdIns(3,4,5)P_3_-3-phosphatase and tumour-suppressor, PTEN inhibits this pathway. p110α, but not other p110s, has a number of onco-mutant variants that are commonly found in cancers. mRNA-seq data shows that MCF10a cells express p110β>>α>δ with undetectable p110γ. Despite this, EGF-stimulated phosphorylation of PKB depended upon p110α-, but not β- or δ- activity. EGF-stimulated chemokinesis, but not chemotaxis, was also dependent upon p110α, but not β- or δ- activity. In the presence of single, endogenous alleles of onco-mutant p110α (H1047R or E545K), basal, but not EGF-stimulated, phosphorylation of PKB was increased and the effect of EGF was fully reversed by p110α inhibitors. Cells expressing either onco-mutant displayed higher basal motility and EGF-stimulated chemokinesis.This latter effect was, however, only partially-sensitive to PI3K inhibitors. In PTEN^−/−^ cells, basal and EGF-stimulated phosphorylation of PKB was substantially increased, but the p110-dependency was variable between cell types. In MDA-MB 468s phosphorylation of PKB was significantly dependent on p110β, but not α- or δ- activity; in PTEN^−/−^ MCF10a it remained, like the parental cells, p110α-dependent. Surprisingly, loss of PTEN suppressed basal motility and EGF-stimulated chemokinesis. These results indicate that; p110α is required for EGF signaling to PKB and chemokinesis, but not chemotaxis; onco-mutant alleles of p110α augment signaling in the absence of EGF and may increase motility, in part, *via* acutely modulating PI3K-activity-independent mechanisms. Finally, we demonstrate that there is not a universal mechanism that up-regulates p110β function in the absence of PTEN.

## Introduction

Phosphoinositide 3-kinases (PI3Ks) are a ubiquitous family of signal transducing enzymes. There are 3 classes of PI3Ks: the class I PI3Ks, relevant here, can be activated by a large variety of cell surface receptors to produce the signaling lipid, phosphatidylinositol (3,4,5)-trisphosphate (PtdIns(3,4,5)P_3_) [1]. It is now clear that PtdIns(3,4,5)P_3_ is a signal that drives recruitment of a family of PI3K effector proteins to the membrane within which it is resident, normally the plasma membrane. The effector proteins typically contain PH domains that can bind with substantial selectivity and affinity to PtdIns(3,4,5)P_3_ and are responsible for conferring their sensitivity to PI3K activation [2]. These effectors contain a number of types of additional homology domains responsible for relaying the PI3K signaling downstream, including; protein serine/threonine kinase (eg protein kinase B (PKB), Phosphoinositide Dependent Kinase-1 (PDK-1)) [3], [4], [5], [6], [7], [8], RhoGAP (Rho-GTPase Activating Proteins) and ArfGAP (eg ARAPs1, 2 and 3) [9], [10], RacGEF (Rac GTPase Guanine nucleotide Exchange Factors) (eg PRex1 and PRex2, Tiam-1)) [11], [12], [13], SH2 (eg DAPP-1) [14], [15], [16] and protein tyrosine kinase (eg BTK, ETK) [17]. Hence class I PI3Ks play a wide ranging role linking activation of receptors to cellular responses such as cell survival (through, eg PKB) [18], [19], [20], cell movement (RhoGAPs and RacGEFs) [7], [21], [22], proliferation (PKB) [23], [24] and secretion [25].

The mechanism by which PtdIns(3,4,5)P_3_ activates effectors was first revealed for PKB [5], [6], [8]. The PH domain of PKB binds PtdIns(3,4,5)P_3_ and this leads to the recruitment of PKB to the plasma membrane. PDK-1, a kinase capable of phosphorylating T308 (numbering based on PKBα sequence) in the activation loop of PKB, is also recruited to PtdIns(3,4,5)P_3_ -containing membranes *via* its PH domain. This co-localisation and a change in the conformation of PKB resulting from PtdIns(3,4,5)P_3_-binding rendering T308 more available leads to a huge increase in the rate of phosphorylation and activation of PKB. Full activation of PKB is achieved by phosphorylation of S473 by the TORC2 (Target Of Rapamycin) complex [26], this event is dependent on class I PI3K activity, possibly because PtdIns(3,4,5)P_3_ can activate TORC2 directly and PtdIns(3,4,5)P_3_ -bound PKB is a better substrate [27]. PKB has a number of important substrates including GSK3β, FOXO transcription factors and TSC2 [28] and these generate impacts in a huge range of cell functions including cell growth, survival and metabolism [29].

There are 4 Class I PI3Ks; they are all heterodimers, made up of a regulatory and a catalytic subunit. The 4 distinct catalytic subunits, p110s α, β, δ and γ, give their names to the heterodimers they form, and are further divided into Class IA (α, β, δ) and IB (γ) on the basis of their mode of regulation and the adaptor subunits they bind. The Class IA PI3Ks bind regulatory subunits from the SH2 domain-containing p85-family of adaptors (derived from 3 genes, p85α, p85β and p55) that bind to protein tyrosine phosphate residues (classically within a YXXM motif). These PI3Ks are recruited by tyrosine kinase-based signaling networks, such as those activated by insulin, EGF and PDGF [1]. The sole Class IB PI3K (p110γ) can bind to 2 related adaptors, p84 or p101, that confer sensitivity to G-protein βγ-subunits [30], [31], [32]. Hence PI3Kγ functions predominantly downstream of G-protein activation [33].

PtdIns(3,4,5)P_3_ signals can be terminated through the action of either phosphoinositide 3- or 5-phosphatase activities (PTEN or SHIP family), the relative significance of these 2 routes depends on the cell type. PTEN appears, however, to be particularly important in controlling PtdIns(3,4,5)P_3_ relevant to cell growth, survival and proliferation in many cell types, as revealed by its status as an important tumour suppressor [34].

A large proportion of human cancers carry mutations in PI3K pathway components, including loss of PTEN, and “activating” mutations in p110α (PIK3CA, these focus into hot-spots at H1047 and E545 [35]), p85α [36] and PKB [37], [38]. These observations demonstrate the potential for chronic activation of the pathway to advantage cancer cells, they do not show that the normal physiological functions such as cell survival, growth or proliferation will be dependent upon the endogenous counterparts of the onco-mutant proteins.

Over the last few years, varieties of PI3K-specific reagents or approaches have revealed that the individual Class I PI3Ks have unique or selective roles in particular cellular contexts. Initial work relied on use of selective anti-catalytic antibodies [24], [39], [40], [41], [42], [43], [44] and more recently PI3K-selective small molecule inhibitors [45], [46], [47], [48], [49], [50], [51], [52], RNAi [53], [54], [55], PI3K KO mice, and cell lines derived from them [56], [57], [58], [59], [60], [61], [62], [63], [64], [65], [66], [67] and analysis of human cancers [35], [68]. This body of work has revealed that individual class I PI3Ks have a complex combination of unique and over-lapping roles depending on the setting. For example, any of the class IA PI3Ks can generate the PtdIns(3,4,5)P_3_ required to support cell survival in the absence of others [69]. It is not clear, however, whether all three isoforms generate a significant amount of PtdIns(3,4,5)P_3_ when all are expressed normally in this setting. Some cell responses have been reported to be uniquely dependent upon a specific class IA PI3K or differentially dependent upon one or more members, despite the fact all three class IA PI3Ks are expressed in the relevant cell type [45], [59], [65], [67]. The molecular explanations of these examples of specificity are not clear but appear unlikely to be explained by differences in expression. It appears that in at least some of these cases of differential dependency of responses on class IA PI3Ks are critically dependent on the strength of the driving stimulus [67]. At maximal levels of activation there is greater redundancy, at low intensity activation there is more evidence of individual isoforms having differential roles.

In an interesting variant of the principle of PI3K isoform specific signaling, there is also evidence that in the genetic absence of PTEN, or possibly in basal, unstimulated cells [45], that PI3Kβ becomes relatively more important in driving the PI3K pathway in several different cell types [45], [70], [71], [72]. The mechanisms underpinning these observations are unclear but therapeutically important.

EGF can drive chemotaxis of many breast-derived cell lines. This process is thought to be relevant to cancer invasion *in vivo*. EGF-stimulated chemotaxis has been shown to be dependent on class I PI3K activity and to be increased by expression of onco-mutant p110α alleles [55]. These results have been considered to result from a role for intracellular gradients of PtdIns(3,4,5)P_3_ in directing the orientation of lamellipodia formation and through that migration [73].

The MDA-MB cultured cell lines are a series of breast cancer-derived cell lines that have become widely used in studies of the cancer cell phenotype. MDA-MB 231 cells have wild-type PIK3CA (at known “hot spots”; Wellcome Trust Sanger Institute Cancer Genome Project) and PTEN loci but an activated Ras allele, MDA-MB 468 are more transformed and have a wild-type PIK3CA locus but have lost PTEN function. These cell lines respond to a number of extracellular stimuli, particularly EGF and insulin, both of which can stimulate PI3K activation in a variety of breast cancer cell lines. EGF has also been demonstrated to drive chemotaxis in MDA-MB 231 cells [74], however, MDA-MB 468 cells high level of transformation appears to correlate with a flattened, highly adherent and relatively immotile phenotype.

Published work already indicates that EGF can stimulate PI3K activation in MDA-MB cells and hence phosphorylation of PKB (Akt) at both Threonine 308 (in the activation loop) and Serine 473 (hydrophobic pocket) [75]. Consistent with the recognized role of PTEN as a physiological antagonist of PI3K signaling MDA-MB 468 cells, or others, that lack PTEN, show very high basal levels of PKB phosphorylation [75], [76]. In addition, it has been shown that EGF-stimulated or basal migration [44], [74], [77] and survival and proliferation of these cells are PI3K dependent [75].

MCF10a cells are a non-transformed, immortalized, human breast epithelial cell line. Through application of homologous targeting strategies a panel of isogenic MCF10a sub-lines have been derived expressing single onco-mutant alleles or unable to express a specific open-reading frame. Examples include PTEN^−/−^, p110α^H1047R/WT^ and p110α^E545K/WT^ [78], [79], [80], [81].

We have addressed the role of class IA PI3K signaling in human breast-derived cell lines MCF10a, MDA-MB 231 and MDA-MB 468 cells using shRNAi, small molecule inhibitors and homologously-targeted, isogenic cell lines.

## Materials and Methods

### Cell Lines and Tissue Culture

MCF10a are non-transformed human breast epithelial cells. PTEN^−/−^, p110α^H1047R/WT^, p110α^E545K/WT^ and PKB^E17K/WT^ MCF10a cell lines were generated by targeted homologous recombination and were obtained from Horizon Discovery LTD together with their parental cell lines.

All MCF10a cell lines are maintained at 37°C with 5% CO2 in DMEM/F12 supplemented with 5% horse serum, 2 ng/ml EGF (except for the p110α^H1047R/WT^, p110α^E545K/WT^ cell lines, no EGF), 10 µg/ml insulin, 0.1 µg/ml cholera toxin, 0.5 µg/ml hydrocortisone, 1% w/v penicillin/streptomycin (P/S). The assay medium used for the starvation, chemokinesis and phospho-PKB assays is made of DMEM/F12 supplemented with 1% charcoal dextran treated foetal bovine serum (FBS), 0.1 µg/ml cholera toxin, 0.5 µg/ml hydrocortisone, 1% P/S.

The MDA-MB 231 and MDA-MB 468 cells were maintained in DMEM and Leibovitz’s L-15 Medium respectively, with 10% FBS and 1% P/S. The Wellcome Trust Sanger Institute Cancer Genome Project defines both of these cell lines as containing no detectable mutations in their PIK3CA (PI3Kα) loci, whilst MDA-MB 468, but not MDA-MB 231, cells are defined as PTEN negative. MDA-MB 231 but not MDA-MB 468 cells carry a K-Ras^G13D^ allele.

293FT human embryonic kidney (HEK) cells were from Invitrogen and were cultured at 37°C with 5% CO_2_ in DMEM with 10% foetal bovine serum, 1% P/S and 0.6 mg/ml of G-418 disulfate (Melford, UK).

DMEM/F12, DMEM, Leibovitz’s L-15 medium, FBS and P/S are purchased from Invitrogen, horse serum and charcoal dextran treated FBS from PAA and the other reagents from Sigma.

### RNAi Oligonucleotide Sequence

siRNA 19-nt mRNA sequence for PI3Kα were obtained from Dharmacon siDESIGN® Center web site (http://www.dharmacon.com/DesignCenter/DesignCenterPage.aspx).

RNAi A1: TGTCTATCCTCCAAATGTA, and RNAi A2: GTATGTTGCTATCCTCTGA. The RNAi control used are: RNAi N1 against firefly luciferase; GTGCGTTGCTAGTACCAAC, RNAi N2: CCCGACTGCTATTCTTTTC, RNAi N3: GTACTCCTAGTTAGTTCAG and RNAi N4: GCTTGGGCGAGAGTAAGTA.

### Constructs for Inducible Expression of shRNAi

A system, based on Doxycycline-induced expression of a Tet-Krab-repressed bis-cistronic element expressing eGFP and an shRNAi construct, developed in the Trono lab was employed [82]. We introduced a number of modifications to the system. Firstly, we introduced a Zeocin-selection cassette in place of ds-Red in the vector tTR/KRAB-Red. IRES-Zeocin sequence was amplified from LZRS-MS-IRES-Zeo/pBR vector by PCR with the oligonucleotides 5′-GCTACGTAAATTCCGCCCCCCCCCCCCTCTCCCTC-3′ and 5′-GACTAGTAAATTCTAGAGTCGCGGCCGTCAGTCCTGCTCCTCGGCCA-3′. The fragment was cut with SnaBI and SpeI before to be integrated into pLV-tTR/KRAB-Red. The new bicistronic vector called pLV- tTR/KRAB-Zeo expressed Tet-Krab and can be selected using Zeocin (InvivoGen, CA, USA).

pLV-TH RNAi. The oligos containing the hairpin RNAi were integrated into pCMS3-H1p-EGFP in order to obtain the oligos-H1p promoter. All constructs were sequenced to ensure integrity. The oligos-H1p-promoter fragment was extracted using EcoRI and ClaI, purified and ligated into an HIV-1 derived lentiviral vector, pLV-TH.

### Lentivirus

The packaging cell line HEK 293FT were seeded at 4.5×10^6^ cells in 5 ml of DMEM, 10% FBS, 1% PS in a TC 90 mm dish (Nunc) the previous day. One hour before transfection the medium was replaced with Opti-MEM®I medium (Gibco/Invitrogen) containing 10% FBS. 6 µg of the transducing vector (pLV-TH with or without the RNAi), 3 µg of the packaging vector pCMV-ΔR8.91 (Trono lab) and 3 µg of the VSVG envelope pMD.G (Trono lab) were co-transfected by LipofectAMINE 2000 (Invitrogen), according to the manufacturer’s instructions. The medium was changed the next day and cells were cultured for another 48 h. The medium, containing the virus, was then collected and cleared of cell debris by centrifugation (250×*g* for 5 min) and filtration (0.45 µm; Millipore). Lentiviral particles were concentrated by: centrifugation of lentiviral supernatants for 2 hours at 4°C at 25000 rpm. The supernatant was discarded and 300 µl of cold PBS was added. Tubes were left overnight at 4°C. The following day, PBS was carefully pipetted 20× over the pellet, after which the PBS containing the virus was snap frozen in liquid nitrogen and stored at −80°C.

### Transduction of Target Cells

MDA-MB 231 and MDA-MB 468 were seeded in each well of a 24-well plate at the concentration of 3×10^4^ cells/ml and incubated at 37°C for 24 h. The transduction was carried out by adding the concentrated virus in the presence of Polybrene (8 µg/ml; Sigma). The virus was removed after 24 h and replaced with medium. To improve the percentage of cells infected by the virus, different concentrations of each virus were tested. The MDA-MB 231 and MDA-MB 468 cells were first transduced with virus containing LV-tTR/KRAB-Zeo. The resulting cells were selected with Zeocin (400 µg/ml) and the selected populations were called MDA-MB 231Z and MDA-MB 468Z, respectively. These selected populations of cells were further transduced with lentivirus containing LV-TH with or without an RNAi. A few days after transduction, Doxycycline (2 µg/ml; Sigma) was added to the cells to de-repress the expression of the shRNAi and eGFP. Transduced cells were analysed by flow cytometry (LSR-II; BD Biosciences) after 4–6 days.

### Chemokinesis Assay

MCF10a cells were seeded on collagen IV-precoated glass in 96 wells plates (BD) at the concentration of 3.5×10^3^ cells/well/200 µl in growth medium and left to adhere for 2 hrs at 37°C. After 2 hours of starvation, the assay medium is replaced by 200 µl of assay medium containing the EGF with or without PI3K inhibitors or DMSO (vehicle for all the inhibitors). The cell migration was recorded by videomicroscopy during 18 h at a frequency of 1 image every 12 mins using the BD pathway 855 (the cells are maintained at 37°C with 5% CO2).

Individual cell are manually tracked and the trajectory were analysed using the Manual Tracking and Chemotaxis plug-ins of ImageJ, respectively.

### Chemotaxis Assay and Migration Assays in a Dunn Chamber

Glass coverslips were coated with 250 µl of Growth Factor Reduced Matrigel Matrix (BD) diluted 1/200. After 2 hr incubation at room temperature the Matrigel was washed twice with PBS. MDA-MB 231Z cells were seeded at a concentration of 4×10^4^ cells/dish/2 ml and allowed to adhere to the Matrigel overnight. The next day the cells were starved for 24 h in serum-free DMEM before being mounted on a Dunn Chemotaxis Chamber (Hawksley Technology). EGF (0, 15, 30 or 60 ng/ml in DMEM) was added to the outer well of the chamber with DMEM alone in the inner well. Cell motility was digitally recorded by video microscopy (Zeiss) using a time-lapse interval of 5 mins for 5 hrs. Cells were tracked manually using Metamorph (Molecular devices), and the trajectories were statistically analyzed using Mathematica (Wolfram research).

### Phospho-PKB Immuno-blotting

3.8×10^5^ cells were seeded in 35 mm dish in the morning, left to adhere for 6 hrs and starved for 18 hrs. Cells are pre-incubated with either the inhibitor(s) or DMSO for 20 minutes then incubated with EGF +/− inhibitors or DMSO for a further 15 mins. The incubations were quenched by washing with ice-cold PBS. Cells were scrapped and lysed in 150 µl of ice-cold lysis buffer (20 mM Tris, pH 7.5; 150 mM NaCl; 1 mM EDTA, pH 7.5; 1 mM EGTA, pH 7.5; 0.1% v/v Triton-X100 supplemented with anti-proteases: 10 µg/ml leupeptine, 10 µg/ml aprotinin, 10 µg/ml antipain, 10 µg/ml pepstatin A, 0.1 mM PMSF and anti-phosphatases: 2.5 mM Na_4_P_2_O_7_, 5 mM β-glycerolphosphate, 1 mM Na_3_VO_4_, 25 mM NaF). After 30 min solubilisation at 4°C with agitation, lysates were centrifuged (13,000 rpm, 10 min, 4°C) and the supernatants collected and diluted in sample buffer.

Proteins (30 µg/well) are resolved on a 10% SDS-PAGE gel, transferred to PVDF membranes and blotted with a mix of primary antibodies; anti-phospho-Akt-S473 (D9E, 4060, Cell Signaling), anti-phospho-Akt-T308 (5106, Cell Signaling) and anti-β-COP (kind gift from Dr Nick Ktistakis, Babraham Institute, UK) for 2 hrs at room temperature. They are then washed in TBS (40 mM Tris/HCl, pH 8.0, 22°C; 0.14 M, NaCl) containing 0.1% v/v Tween 20 and incubated with a mix of Infrared Dye coupled secondary antibodies (IRdye 680 goat anti-rabbit IgG, IRdye 800 goat anti-mouse IgG, Li-Cor). The membranes are imaged with the Li-Cor Odyssey Infrared Imaging System using the 700 nm and 800 nm channels. Signals were quantified using the Image Studio software.

Alternatively, 8×10^6^ MDA-MB 231Z or MDA-MB 468Z cells induced with Dox or its vehicle (see above) and potentially expressing irrelevant or specific shRNAi constructs were seeded in 5 ml DMEM, 10% FBS, 1% PS in a 90 mm dish and incubated overnight. The cells were starved in DMEM for 20 hrs before being stimulated either with EGF (4 ng/ml for the 231Z cells and 0.1 ng/ml for the 468Z cells) for 5 min. For the control the cells were treated with LY294002 (30 µM) for 15 min. The cells were washed with cold PBS and lysed with 300 µl lysis buffer (20 mM Tris, pH 7.5, 4°C; 150 mM NaCl; 1 mM EDTA; 1 mM EGTA; 1% Triton X-100; 2.5 mM Sodium Pyrophosphate; 1 mM β-glycerol phosphate; 1 mM Na_3_VO_3_; 1 µg/ml leupeptin; 1 mM PMSF). After centrifugation 100 µl of the supernatant was snap frozen in liquid nitrogen and kept at −80°C, the remaining sample was used to determine the concentration of protein using a Bradford protein Assay (BioRad). The appropriate amount of protein was loaded and separated in a 10% SDS–PAGE gel, transferred to nitrocellulose and blotted with either an anti-Phospho-Akt-T308 or an anti-Phospho-Akt-S473. The membrane was washed and incubated with an HRP-conjugated, goat anti-mouse or -rabbit antibody preparation (BioRad). Signals were detected using the ECL detection system.

### PI3K Inhibitors

TGX221, PI103 (Cayman); PIK75 (Axon Medchem); LY294002 (Sigma); A66, IC87114 (Selleckchem).

## Results

EGF has been shown to stimulate activation of class IA PI3Ks and phosphorylation and activation of PKB in breast-derived cell lines. Previous work has indicated this response can be reduced by about 50% by shRNAi suppression of endogenous p110α and over-expression of a non-silenced, kinase-dead p110α allele in MDA-MB 231 cells [55]. In other work, a partially selective p110α inhibitor (PIK75) reduced phosphorylation of PKB in serum-stimulated MDA-MB 231 and 468 cells weakly but in serum-stimulated MCF10a cells strongly [83]; whilst a highly selective p110α inhibitor (A66) only partially reduced phosphorylation of PKB in insulin-stimulated MCF7 cells [51].

**Figure 1:**
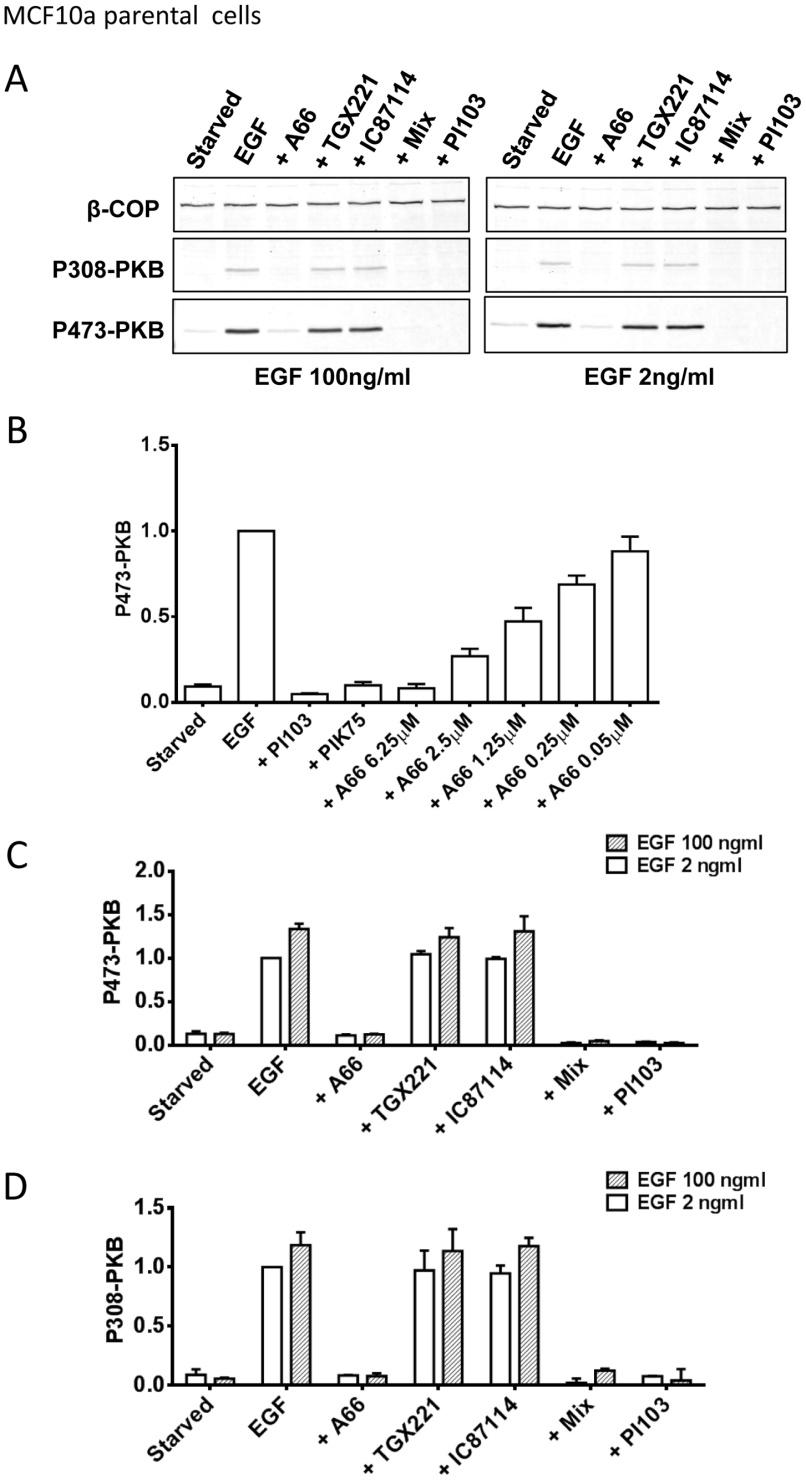
PI3Kα is required for EGF-stimulated PKB phosphorylation in MCF10a cells. MCF10a cells were serum-starved, pre-incubated with inhibitors or vehicle for 20 mins and stimulated with EGF (at the indicated doses) or its vehicle (the vehicle of the inhibitors was only was added to those samples stimulated with EGF without inhibitors or “starved”). After 15 mins the cells were lysed, aliquots were immuno-blotted with anti-β-COP (loading control, 110 kD), anti-phospho-T308-PKB and -S473-PKB antibodies simultaneously on the same filters. The immobilized antibodies were quantified with fluorescent 2° antibodies (goat-anti mouse-IRDye 800 for T308 and β-COP and goat-anti rabbit-IRDye 680 for S473 and a Li-Cor image analysis platform. Data are presented normalized to β-COP expression in the same sample. **Panel A**. shows a representative immuno-blot used to derive data shown in C and D. The final concentrations of the inhibitors with the cells were; A66, 6 µM; TGX221, 40 nM; IC87114, 1 µM; “mix”: A66, 6 µM+TGX221, 40 nM+IC87114, 1 µM; PI103, 1 µM. **Panel B**. The conditions of the experiments and the phosphorylation of S473-PKB was quantified, as in A (except, the experiment included PIK75 at 1 µM and the concentrations of A66, in µM, shown). The data are means ± SE (n = 3 experiments). The data indicate an IC50 of 800 nM. **Panel C**. The conditions of the experiments and the phosphorylation of S473-PKB was quantified, as in A. The data shown are means ± SE (n = 3 experiments). **Panel D**. The conditions of the experiments and the phosphorylation of T308-PKB was quantified, as in A. The data shown are means ± SE (n = 3 experiments).

We found that in MCF10a cells use of the selective p110α inhibitor A66 [51] lead to substantial, greater than 90%, and potent (IC50 approx 800 nM) inhibition of sub-maximal-EGF-stimulated phosphorylation of T308 and S473-PKB (Fig. 1). These results were similar to those generated with a potent inhibitor of PI3Kα, PIK75. However the effects of this compound may be unspecific [51] and we found it potently inhibited the growth and metabolism (see Fig. S1) and movement (not shown) of MCF10a and MDA-MB cell types in a manner that suggested it has toxic off-target effects. A pan-class IA PI3K inhibitor PI103 (Fig. 1) also inhibited EGF-stimulated phosphorylation of PKB. Unlike PIK75 and PI103, A66 had small or no effect on the growth and metabolism of MCF10a or MDA-MB type cells (Fig. S1), suggesting that its effects on PKB were unlikely to be *via* indirect effects on the cell cycle or survival. In contrast, IC87114 (p110δ-selective up to 2 µM) and TGX221 (p110β selective up to about 40–60 nM) had no significant effect on these responses. These results suggest that p110α-containing PI3Ks (PI3Kα) are required for EGF-stimulated activation of PKB in MCF10a cells and this role is not redundant with other class IA PI3Ks.

**Figure 2:**
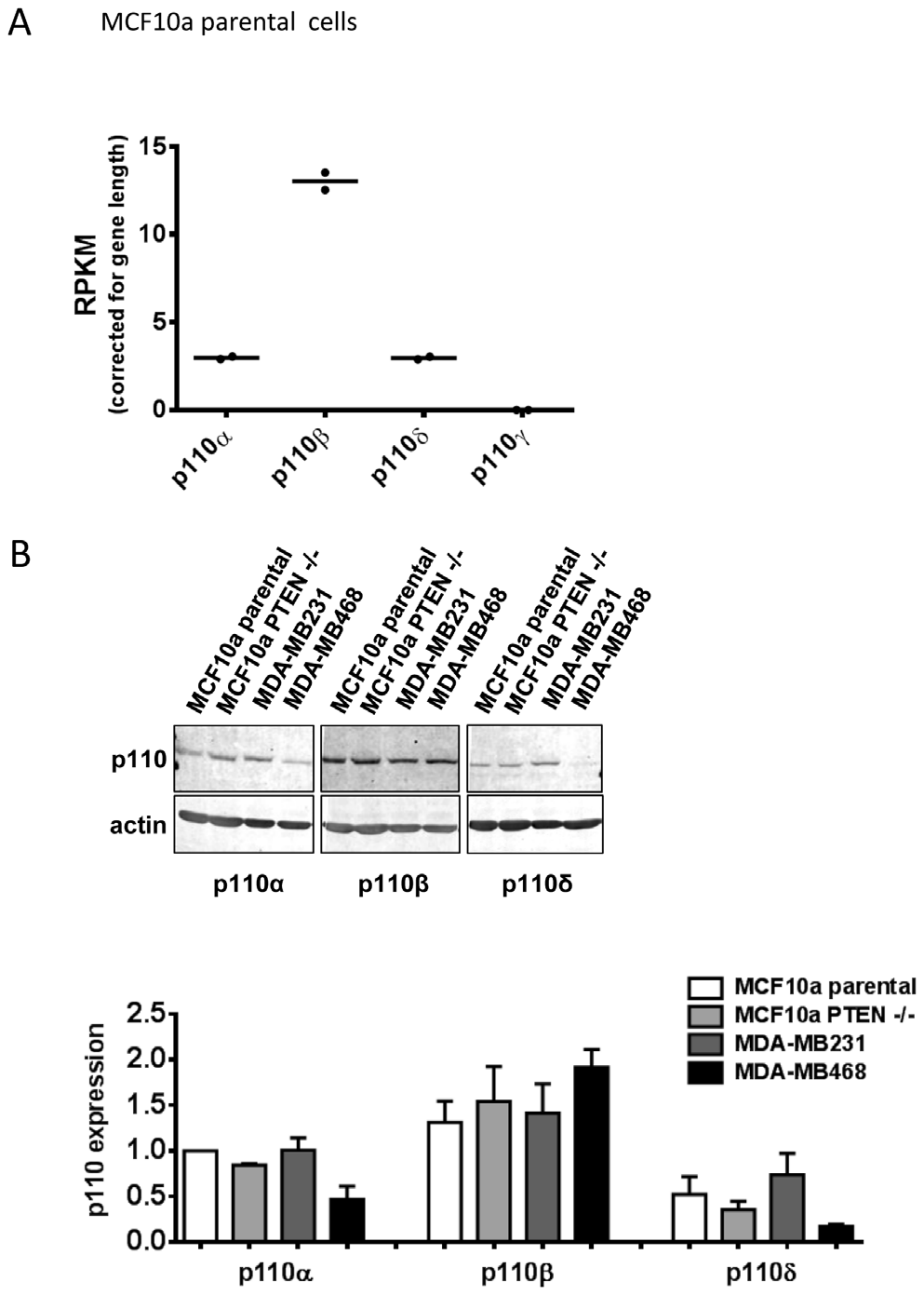
Relative expression of class I PI3Ks in breast-derived cell lines. **Panel A**. MCF10a cells were cultured in full growth medium and then lysed and processed to perform mRNA-seq analysis. The corrected number of mapped sequence reads from p110α, p110β, p110δ and p110γ mRNAs are presented in a scatter plot based on 2 independent preparations of mRNA. **Panel B**. The indicated cells were grown in full growth medium and lysed and processed to quantify their relative expression of p110α, β and δ by immuno-blotting and use of fluorescent 2° antibodies and Li-Cor imaging. Actin was used as a loading control. The upper part of the figure shows a representative immuno-blot used to compile the data shown in the lower part of the figure. The data presented in the graph are means ± SE (n = 3 experiments).

mRNA-seq analysis of growing MCF10a cells (in the presence of EGF) revealed that the relative number of molecules of p110β mRNA was substantially greater than that of p110α and then p110δ (p110γ mRNA was not detected) (Fig. 2). These observations are corroborated by immuno-blots of MCF10a and related cell lines with anti-p110α, p110β and p110δ antibodies indicating all three isoforms can be readily detected in these cell types (Fig. 2). In the context of evidence that expression of class I PI3K subunits mRNAs correlated very strongly with the relative abundance of the respective proteins [84], this suggested that p110β is the most abundant class I PI3K catalytic subunit in these cells.

Given the powerfully-argued view that p110α is the least basally active of the class IA catalytic subunits [85], the implication of these results is that in MCF10a cells PI3Kα is preferentially sensitive to EGF stimulation.

**Figure 3:**
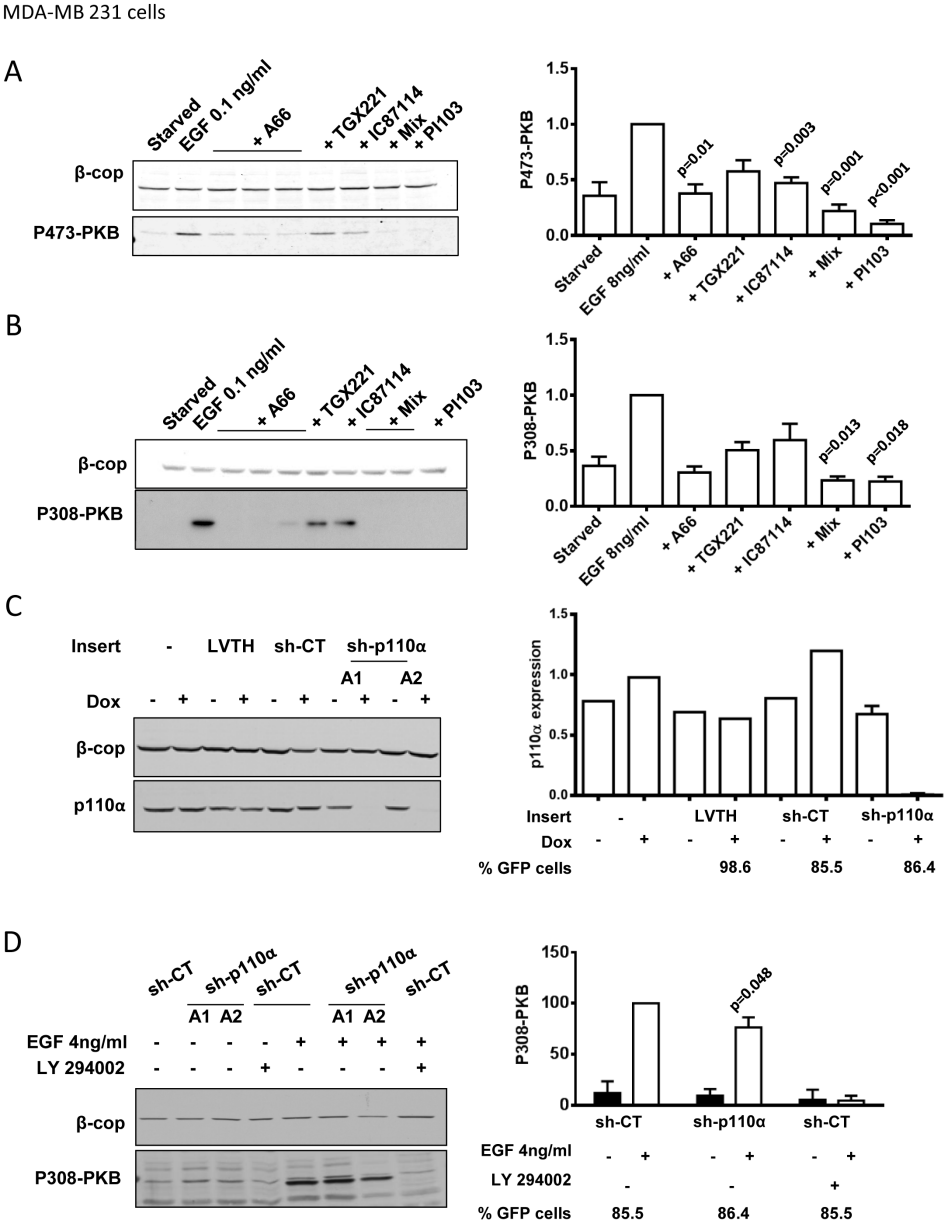
Class IA PI3K-dependent regulation of PKB phosphorylation in MDA-MB 231 cells. MDA-MB 231 cells, or lentivirus-transduced derivative cultures, including those expressing Dox-inducible shRNAi constructs, were serum-starved, pre-incubated with inhibitors or vehicle for 20 mins and then stimulated with EGF (at the indicated dose) or its vehicle (“starved”). Phosphorylation of PKB was quantified by immuno-blotting with fluorescent or HRP-linked 2° antibodies. **Panel A.** Shows, on the left, a representative immuno-blot of β-COP and S473-PKB in the same, MDA-MB 231 cell-derived, samples under the conditions indicated. The final concentrations of the inhibitors on the EGF-stimulated cells were as shown (mix, A66, 6 µM+TGX221, 40 nM+IC87114, 1 µM). On the right S473-PKB/β-COP signals were expressed relative to the EGF-stimulated, inhibitor-free samples. The concentrations of inhibitors were the same as in the figure, except that; A66 was 6 µM. Data are presented as means ± SE (n = 3–4 experiments). The p values of comparisons between an EGF+inhibitor treatment and the EGF-alone treatments are shown (One-Sample t-test followed by Dunn-Sidak correction for multiple comparisons). **Panel B.** Shows data for the phosphorylation of T308-PKB in the same experiments as in panel A. T308-PKB immuno-blots were quantified using 2°-antibodies linked to HRP and ECL-detection. Final concentrations of inhibitors on the cells were like those in panel A, except the “mix” either contained 6 µM A66 (left) or 2 µM A66 (right). The data presented on the right were calculated as in panel A. The data are means ± SE (n = 3 experiments). **Panel C.** MDA-MB 231 cells or Lentivrus transduced derivatives expressing tet-Krab repressor and plus an additional vector capable of doc-inducible expression of shRNAi (either directed to human p110α (A1 or A2), or irrelevant sequence controls, sh-CT (N1 throughout this figure) plus bis-cistronic eGFP, were used in these experiments. LVTH control cells express tet-Krab and, in the presence of Dox, eGFP but no shRNAi. After 4 days with Dox or vehicle, aliquots of cells were either analysed by FACS, to reveal the % expressing eGFP or by immuno-blotting to quantify expression of p110α. A representative blot, with β-COP as a loading control, is shown on the left. p110α expression normalized to β-COP is quantified from a similar experiment on the right. **Panel D.** Using MDA-MB 231 derivative cell lines as described in C we tested shRNAi directed against p110α on EGF-stimulated phosphorylation of T308-PKB. Cells were starved, pre-incubated with LY294002 (LY) or vehicle for 20 mins then stimulated with EGF (4 ng/ml) (open bars) or vehicle (solid bars) for 15 mins. A representative immuno-blot is shown on the left. Signal from phospho-T308-PKB normalized to β-COP was expressed as a % of the signal in EGF-stimulated control-shRNAi-expressing cells, as shown on the right. Data for p110α-directed or irrelevant shRNAi constructs were pooled to provide an overall comparison. The data are means ± SE (n = 3 experiments). The p-value between was calculated with one-Sample t-test.

In MDA-MB 231 cells, EGF-stimulated phosphorylation of PKB was substantially inhibited by selective inhibitors of PI3Kα. However, selective inhibitors of PI3Ks β and δ reduced the response and mixtures of PI3Ksα, β and δ inhibitors and pan-class IA inhibitors both inhibited the response to a greater extent than PI3Kα inhibition alone (Fig. 3). These results suggest some involvement of other class IA PI3Ks in these responses. Inducible expression of shRNAi specifically directed against p110α in MDA-MB 231 cells also inhibited phosphorylation of PKB (Fig. 3). The extent of inhibition achieved with shRNAi was significantly lower than that seen in the presence of A66 despite the fact the constructs reduced p110α expression by 80–90% (Fig. 3). These results emphasize that individual class IA PI3Ks can be present in apparent excess over the sensitivity of their molecular targets and therefore require greater extents of inhibition to reduce class IA PI3K-dependent signaling. The results also indicate that there are greater roles for PI3Ks β and δ and greater redundancy between class IA PI3Ks in MDA-MB 231 cells than in MCF10a cells.

**Figure 4:**
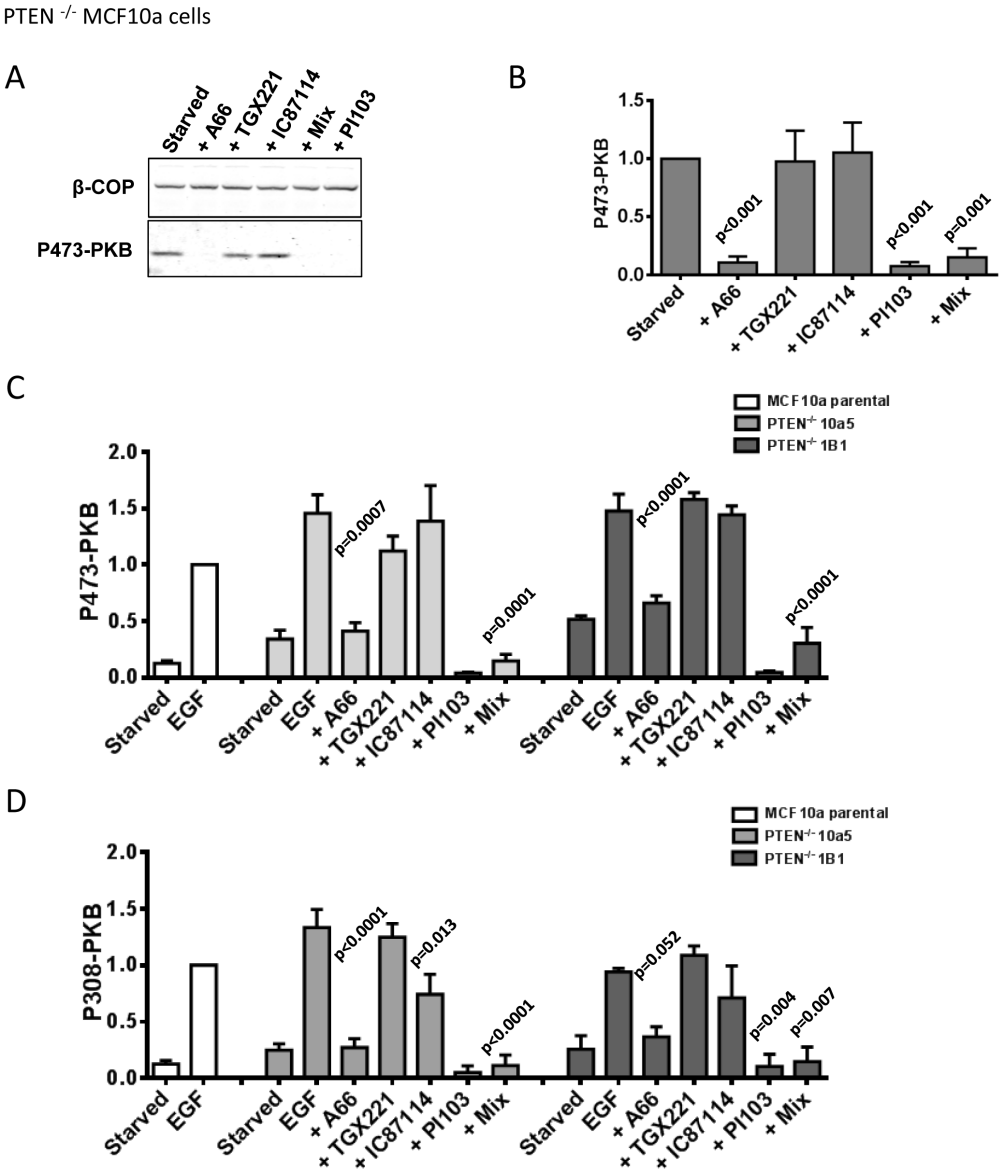
PI3Kα remains the dominant class IA PI3K required for EGF-stimulated PKB phosphorylation in PTEN^−/−^ MCF10a cells. Panel A. PTEN^−/−^ MCF10a cells were serum-starved and then treated with inhibitors or vehicle for 20 mins before phosphorylation of S473-PKB was quantified by immuno-blotting. The final concentrations of inhibitors on the cells were; A66, 6 µM; TGX221, 40 nM; IC87114, 1 µM; “mix” A66, 6 µM+TGX221, 40 nM+IC87114, 1 µM; PI103, 1 µM. A representative immuno-blot is shown. **Panel B.** Data from experiments like those in panel A were normalized for input using β-COP as a loading control and expressed as a proportion of the vehicle-only treated controls. Data presented are means ± SE (n = 3 experiments). Statistical comparisons were conducted as in Fig. 3. **Panel C.** PTEN^−/−^ MCF10a cells (either clone 10a5 or 1B1) were serum-starved, treated with inhibitors or vehicle for 20 mins before stimulation with EGF (2 ng/ml) or vehicle for 15 mins, phosphorylation of S473-PKB was quantified by immuno-blotting. Data presented are means ± SE (n = 3, experiments). The p-values were obtained with a Dunnett test. Panel D. The data for phosphorylation of T308-PKB from the same blots as those shown in panel C.

**Figure 5:**
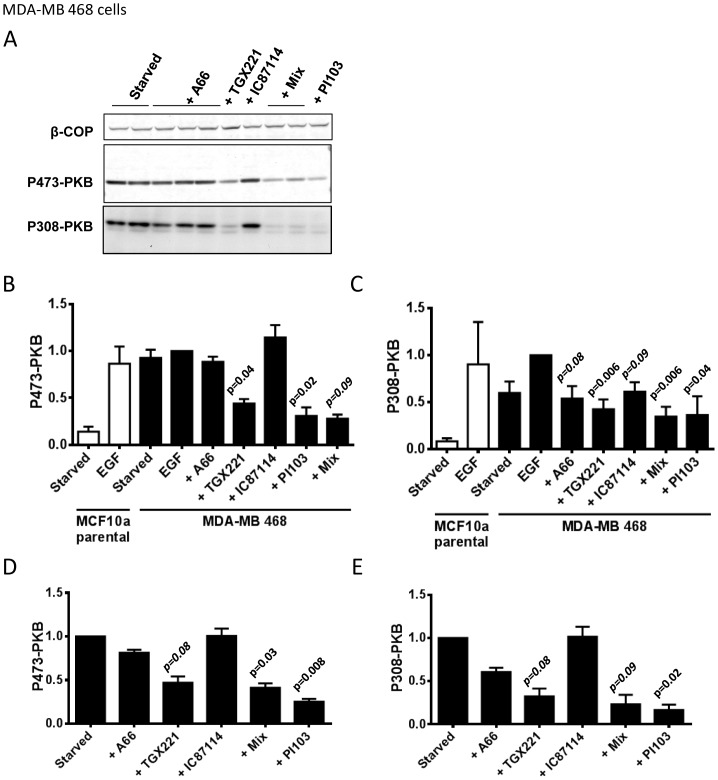
PI3Kβ is the dominant class IA PI3K required for EGF-stimulated PKB phosphorylation in MDA-MB 468 cells. Panel A. MDA-MB 468 cells were serum-starved, treated with inhibitors (inhibitor concentrations, from the left, were; A66, 6 µM; A66, 2 µM, A66, 0.5 µM; TGX221, 40 nM; IC87114, 1 µM; “mix”, A66, 6 µM+TGX221, 40 nM+IC87114, 1 µM; “mix”, except with 2 µM A66; PI103, 1 µM) or vehicle for 20 mins and phosphorylation of PKB was quantified by immuno-blotting (S473 using fluorescent 2°-antibodies, T308 using HRP-linked 2°-antibodies). A representative set of blots, with β-COP as a loading control, are shown. **Panel B.** Data from experiments like those in panel A measuring phosphorylation of S473-PKB were quantified (only data from experiments with 6 µM A66 both alone and as a part of the “mix” are shown) and normalized to the vehicle treated samples (“starved”). Data presented are means ± SE (n = 3, experiments). Statistical comparisons were conducted as in Fig. 3. **Panel C.** Phosphorylation of T308 was quantified with the same blots as those shown in panel B. **Panel D.** Experiments like those shown above except some cells were stimulated with EGF (0.1 ng/ml) or EGF plus inhibitors. Phosphorylation of S473-PKB was quantified by immuno-blotting, normalized for loading using β-COP and corrected between experiments by expressing the data as a proportion of samples that were EGF-stimulated but not treated with inhibitors. Data are presented as means ± SE (n = 3, experiments). **Panel E.** Phosphorylation of T308-PKB was quantified with the same blots as those shown in panel D.

Loss of PTEN is known to substantially increase basal and receptor-stimulated phosphorylation of PKB. MCF10a cells have been subjected to homologous gene targeting techniques to generate clonal, isogenic PTEN^−/−^ cell lines [79]. We employed 2 different clones of these cells (10a5 and 1B1). We found that phosphorylation of T308 and S473-PKB was substantially increased in both PTEN^−/−^, compared to PTEN^+/+^, lines under both basal and EGF-stimulated conditions (Fig. 4). Use of selective inhibitors revealed that phosphorylation of PKB in these cells, like the parental MCF10a cells, was most strongly affected by inhibition of PI3Kα; with EGF-stimulated levels being returned near to those of unstimulated controls and basal, serum-starved levels being reduced still further (Fig. 4). Importantly, for discussion below, selective inhibition of PI3Kβ alone had no effect either under EGF-stimulated or basal conditions. MDA-MB 468 cells do not express PTEN and display relatively very high basal phosphorylation of PKB. Interestingly, both basal and EGF-stimulated phosphorylation of PKB was most effectively reduced by inhibition of PI3Kβ alone and slightly more so by pan-class IA PI3K inhibition (Fig. 5). These results indicate the balance of basal and EGF-stimulated PI3K activities in MDA-MB 468 cells, with a particularly prominent role for PI3Kβ, are quite different to MCF10a and more similar to that seen in MDA-MB 231 cells. It is interesting to note that MDA-MB 468 cells express relatively more p110β compared to the other p110s, compared to MCF10a and MDA-MB 231 cells (Fig. 2, the relative p110β/(p110α+p110δ) signals for MDA-MB 468 cells are 3.0 compared to 0.82 for MDA-MB 231 cells and 0.83 for parental MCF10a cells). Together these results indicate that class IA PI3K signaling is not hard-wired in a manner that leads to PI3Kβ being dominant either in the absence of PTEN or in basal cells.

**Figure 6:**
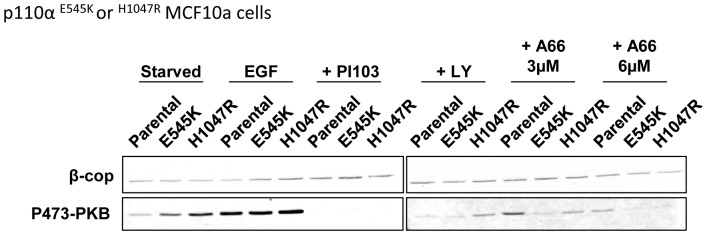
Regulation of PKB phosphorylation in the context of signle activating alleles of p110α in MCF10a cells. MCF10a cells (parental, expressing p110α^E545K/WT^ or p110α^H1047R/WT^) were serum-starved, pretreated with inhibitors or vehicle for 20 mins and stimulated with EGF (2 ng/ml) or vehicle and phosphorylation of S473-PKB was quantified by immuno-blotting using fluorescent 2°-antibodies. β-COP in the same samples was used as a loading control. An experiment, representative of two is shown.

Knock-in of single alleles of onco-mutant H1047R or E545K-p110α into one of the endogenous p110α loci in MCF10a cells [86] increased basal but not sub-maximal EGF-stimulated phosphorylation of PKB (Fig. 6). EGF-stimulated phosphorylation of PKB in H1047R and E545K-expressing cells was fully reversed by selective inhibition of PI3Kα (Fig. 6).

**Figure 7:**
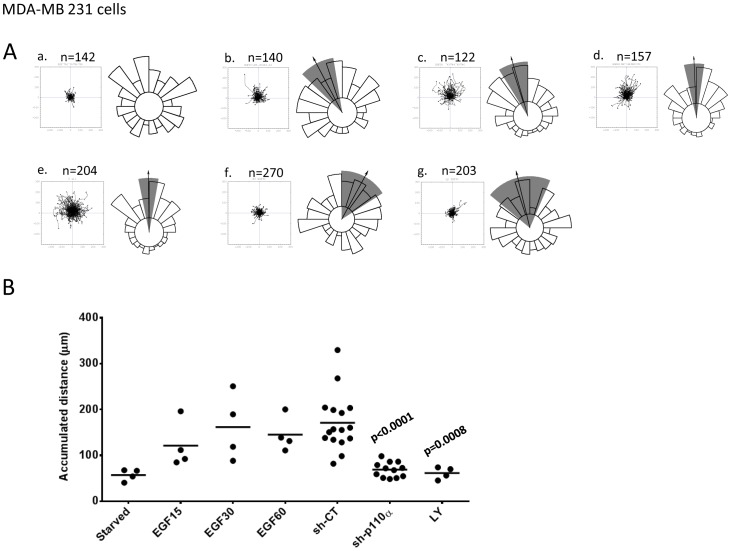
Role of class IA PI3Ks in EGF-stimulated chemokinesis in MDA-MB 231 cells. Panel A. Individual MDA-MB 231 cells were tracked moving on Matrigel in stable gradients of EGF (the concentration of EGF in the reservoir was 0 ng/ml (a), 15 ng/ml (b), 30 ng/ml (c) and 60 ng/ml (d)) or expressing sh-CT (e), sh-p110 (f) or treated with LY294002 (g) in Dunn chambers. The data are presented centre-zeroed with the source of EGF at the top. Directionality was analysed using Mathmatica and significant directionality is denoted by a grey vector and arrow. The number of individual tracks analysed is shown (n) and were collected from at least 3 independent experiments. **Panel B.** The total accumulated distances moved by individual cells in the experiments shown in panel A are shown, the data presented are means. Parental MDA-MB 231 cells (some parental cells were pretreated with 10 µM LY294002) or derivatives expressing either control or p110α-directed shRNAi constructs (3 sh-CT (N2, N3 and N4, see Methods, separate cell lines expressing each construct were used and the data derived were pooled for presentation as they were indistinguishable) and 2 sh-p110α -expressing (A1 and A2, 2 cell lines expressing the individual constructs were used and the data from were pooled for presentation) independent, selected populations which were in the range 80–90% eGFP +ve) were exposed to EGF gradients (30 ng/ml EGF in the reservoir) in Dunn chambers. The cell tracks and their directionality are shown as in panel A. The number of cells tracked (n) is indicated and were collected from at least 4 independent experiments. The total accumulated distances moved by individual cells in the experiments shown in panel A are shown, the data presented are means. The data for all control shRNAi constructs and all p110α-directed constructs were pooled separately to enable an overall comparison of their effects. Statistical comparisons were conducted as in Fig. 4C.

**Figure 8:**
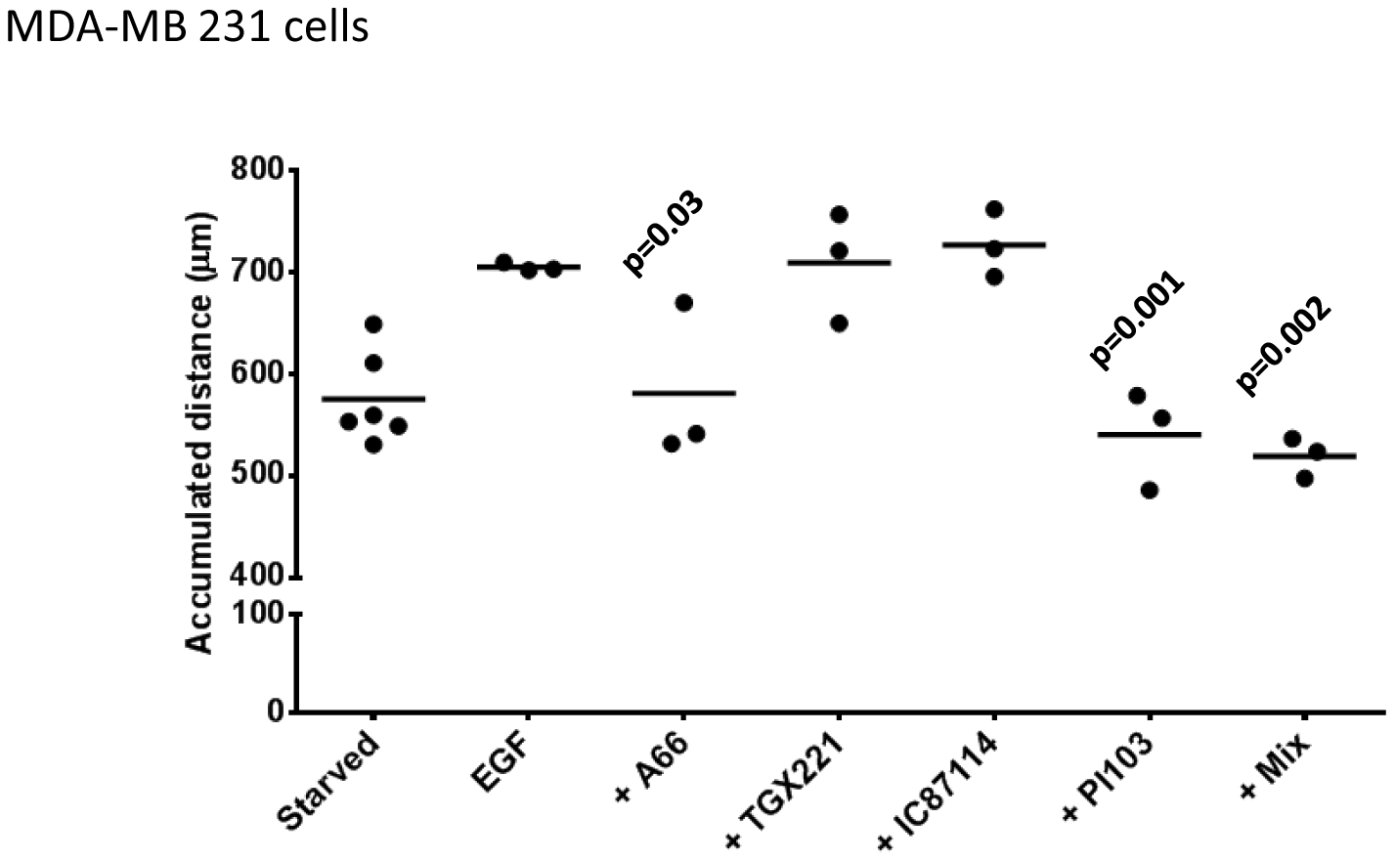
Role of class IA PI3Ks in EGF-stimulated chemokinesis in MDA-MB 231 cells. MDA-MB 231 cells were plated onto collagen IV, pretreated with inhibitors (A66, 6 µM; TGX221, 40 nM; IC87114, 1 µM; “mix”, A66, 6 µM+TGX221, 40 nM+IC87114, 1 µM; PI103, 1 µM) or vehicle (“starved” and EGF alone) and stimulated with EGF (8 ng/ml, uniform) or vehicle (“starved”). Individual cell movement was tracked and the total accumulated distance moved by the cells is presented, the data from at least 3 independent experiments is combined. Statistical comparisons were conducted as in Fig. 3.

**Figure 9:**
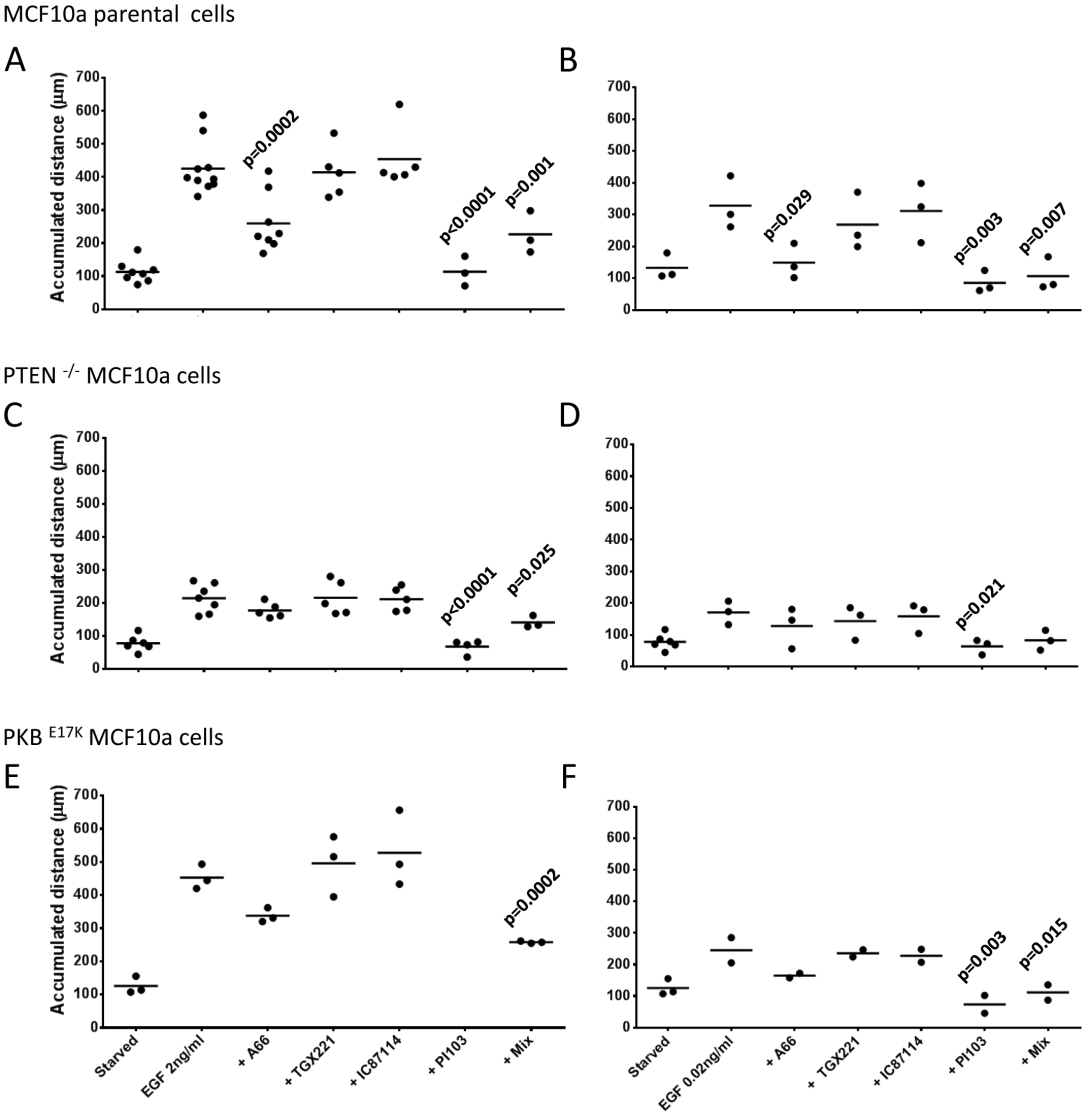
Role of class IA PI3Ks in EGF-stimulated chemokinesis in MCF10a cells. Parental MCF10a cells (A and B), PTEN^−/−^ MCF10a cells (C and D) and PKB^E17K/WT^ MCF10a cells (E and F) were plated on collagen IV, pretreated with inhibitors (A66, 6 µM; TGX221, 40 nM; IC87114, 1 µM; “mix”, A66, 6 µM+TGX221, 40 nM+IC87114, 1 µM; PI103, 1 µM) and stimulated with EGF (uniform, 2 ng/ml, A, C, E and 0.02 ng/ml, B, D and F) or vehicle (“starved”). Individual cells were tracked and the total accumulated distance moved is shown as a mean from at least 3 independent experiments. Statistical comparisons were obtained with a One-Way ANOVA followed by a Dunnett test.

EGF has been shown to stimulate chemokinesis and chemotaxis of a number of cell types, including the MDA-MB cell lines, in a PI3K-dependent fashion [87]. We tracked the movement of individual cells in matrigel-coated surfaces in Boyden chambers within stable gradients of EGF (Fig. 7). These experiments revealed that the MDA-MB 231 cells responded both chemokinetically (moved faster) and chemotactically (moved up a concentration gradient) to EGF. In these types of experiments, pan-PI3K inhibitors substantially reduced the chemokinetic response to EGF (Fig. 7). The chemotactic response to EGF was only apparently weakened because the cells moved less and thus demanded more measurements to achieve statistically-validated chemotaxis. When sufficient individual cells were tracked in the presence of pan-PI3K inhibitors it was clear that although they moved substantially smaller distances, that movement was chemotactic (Fig. 7). Suppression of p110α with inducible shRNAi or pretreatment with PI3Kα-selective, but not PI3Kβ- or PI3Kδ-selective, inhibitors reduced EGF-stimulated chemokinesis but not chemotaxis. These results were not a simple product of the assay format as very similar patterns of results were obtained with different liganded surfaces (collagen IV, upon which unstimulated cells moved substantially further) and experimental designs (eg stimulation with uniform application of EGF in tissue culture dishes) (Fig. 8). Collectively these results suggest that PI3Kα has a preferentially important role in control of MDA-MB 231 cell chemokinesis to EGF. These results were supported by experiments with MCF10a cells stimulated by uniform application of EGF. We found that PI3Kα-selective or pan-class I PI3K inhibitors, but not PI3Kβ- or PI3Kδ-selective inhibitors, reduced EGF-stimulated chemokinesis (Fig. 9). The level of inhibition achieved by PI3Kα inhibitors was greater at lower, sub-maximal doses of EGF, PI3Kβ- and δ- inhibitors, however, remained without affect (Fig. 9).

PTEN^−/−^ MCF10a showed substantially reduced basal and EGF-stimulated chemokinesis (Fig. 9). Although it was clear that chemokinesis in PTEN^−/−^ MCF10a cells was sensitive to PI3Kα-selective and pan-class I PI3K inhibitors the reduced scale of the responses made it impossible to be precise about the extents of inhibition. In keeping with these observations we found that PTEN-negative MDA-MB 468 cells also failed to move sufficiently to reliably measure their chemokinetic or chemotactic responses to EGF (not shown).

Knock-in of a single onco-mutant allele of PKB in MCF10a cells had no effect on basal or EGF-stimulated chemokinesis (Fig. 9).

**Figure 10:**
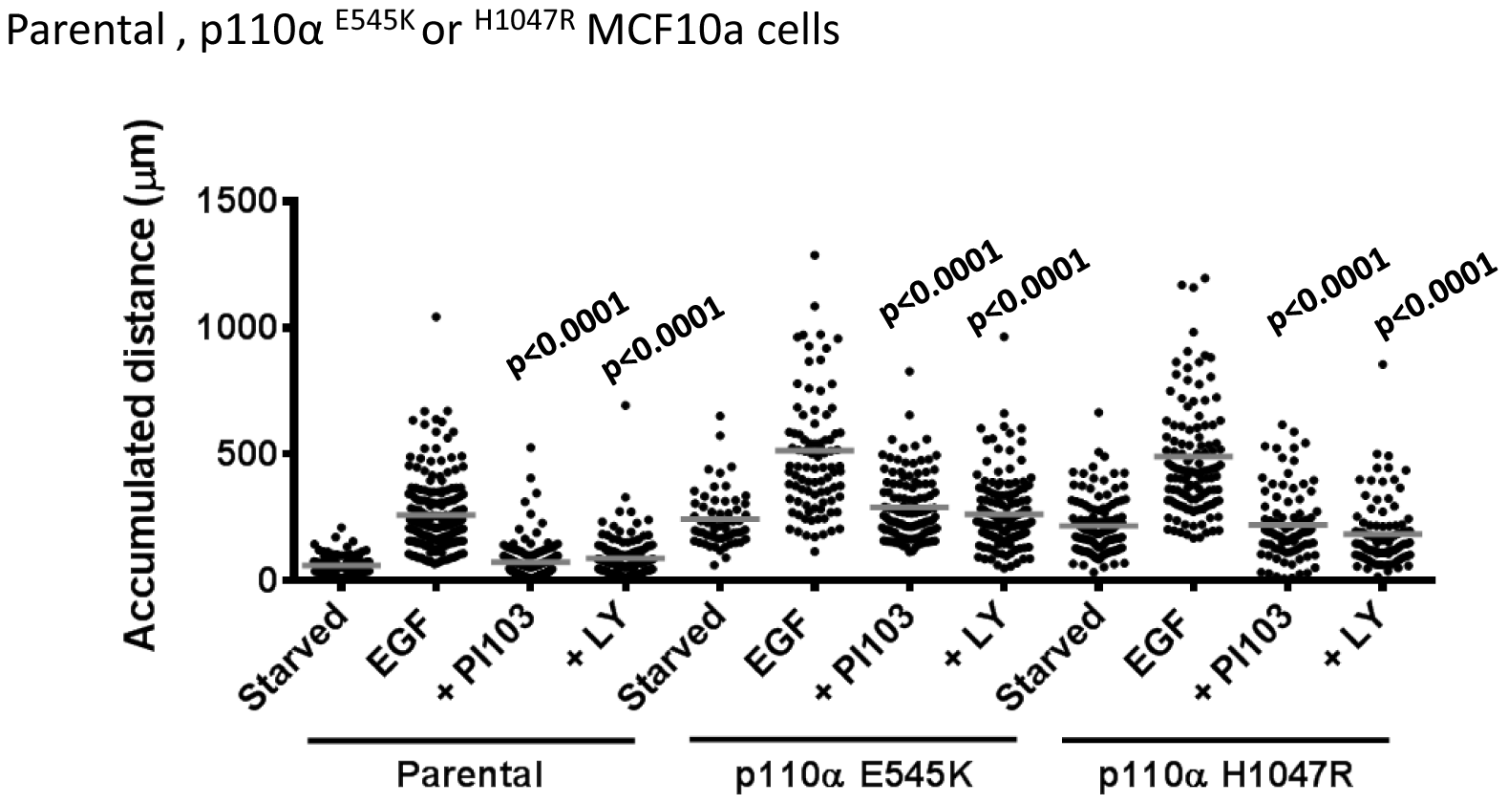
Role of class IA PI3Ks in EGF-stimulated chemokinesis in MCF10a cells. Parental MCF10a, p110α^E545K/WT^ or p110α^H1047R/WT^ cells were treated exactly as described in Figure 9 to measure their chemokinetic responses to EGF (uniform, 2 ng/ml) in the presence or absence of PI3K inhibitors (PI103, 1 µM; LY294002, 20 µM). The total accumulated distance individual cells moved are shown with their relevant means. Statistical comparisons were made between samples treated with EGF alone and those with EGF+inhibitors for each cell type. Statistical comparisons were obtained with a One-Way ANOVA followed by a Tukey test.

Finally, knock-in of single onco-mutant alleles of p110α into MCF10a cells substantially increased basal and EGF-stimulated chemokinesis (Fig. 10). Notably, although PI3K inhibitors reduced EGF-stimulated phosphorylation of PKB well below the levels seen in serum-starved p110α-onco-mutant-expressing cells (Fig. 6), the same inhibitors did not reduce EGF-stimulated chemokinesis equivalently.

## Discussion

Previous work has shown that class IA PI3Ks can have both unique and redundant functions [39], [69], [88]. We have found that PI3Kα has a unique and major role in EGF-stimulated phosphorylation of PKB in MCF10a cells. In MCF10a cells both mRNA-seq and immuno-blots indicate that p110α is not differentially highly expressed compared to related cell types; indeed, these results suggest p110β is likely the most abundant of the class IA PI3Ks in all of these cell types. Furthermore, recent insights into the structure/function of the class IA p110s suggest that p110α is the least basally active of the catalytic subunits and this probably results from it displaying a distribution of conformations that does not favour membrane association and lipid phosphorylation. Onco-mutations, that appear most commonly in p110α, or receptor activation probably act to increase the proportion of p110α in a catalytically activated conformation. These factors suggest it is very unlikely that PI3Kα is dominant in EGF-stimulated phosphorylation of PKB because it is the “most active” or the most abundant class IA PI3Ks. It seems more likely that it is because PI3Kα is activated preferentially by EGF signaling. The simplest explanations for this are (1) increased recruitment/activation by EGFR; (2) increased sensitivity to activated Ras (although this latter possibility would seem unlikely given that in the presence of an activated Ras allele in MDA-MB 231 cells PI3Kα is apparently less important, see below).

In MDA-MB 231 cells PI3Kα has an important and unique role in EGF-stimulated phosphorylation of PKB, however, inhibition of other class IA PI3Ks alone had significant effects and pan-class I PI3Ks inhibitors had bigger effects than PI3Kα-selective inhibitors. Given current knowledge of the regulation of p110s by Ras-family GTPases; that p110α and p110δ, but not p110β [89], are probably regulated by Ras GTPases; it is difficult to explain this changed pattern by the imposition of an activated Ras allele alone. Further, the differential expression of the p110s (Fig. 2) between cell types, with MCF10a and MDA-MB 231 cells being broadly similar, and our argument above, suggesting it is unlikely to be important in determining that p110α is dominant in MCF10a cells, seems unlikely to explain this change.

We found that phosphorylation of PKB in basal MDA-MB 468 cells was very high, in keeping with their loss of PTEN, and EGF had little effect on top of that background. In both basal and EGF-stimulated MDA-MB 468 cells PKB phosphorylation was most sensitive to PI3Kβ-selective inhibitors. A number of studies have described preferential roles for PI3Kβ emerging in the absence of PTEN [70], [72], [90] and molecular explanations for this, based on the apparent association between PTEN and p85 [91] possibly occurring preferentially with p110β-containing p85:p110 complexes [71], [92] and leading to PTEN repressing PI3Kβ function selectively, have been put forward. We found, however, that targeted loss of PTEN in otherwise isogenic MCF10a cells did not change the dominant role for PI3Kα in either basal or EGF-stimulated phosphorylation of PKB. The implication of this result, given our data indicating p110β is expressed in MCF10a cells and that is unchanged by loss of PTEN (Fig. 2), is that there are no hard-wired molecular contacts that mean loss of PTEN will always lead to PI3Kβ becoming functionally dominant. This view suggests that the reason why PI3Kβ is more important in MDA-MB 468 cells is, at most, weakly connected to their PTEN status. It is notable that MDA-MB 468 cells express relatively more p110β compared to the other class IA PI3K catalytic subunits than the other cell lines we have studied, however, given our observations above it seems unlikely that this alone is sufficient to explain this phenotype.

Knock-in of single onco-mutant alleles (H1047R or E545K) of p110α into MCF10a cells, in parallel with a single normal p110α allele, lead to an increase in basal but not EGF-stimulated phosphorylation of PKB. This type of result has been described previously and is presumably, at least partly, the reason why these appear to be driver mutations in cancer. However, phosphorylation of PKB in these cells could be completely suppressed with pan class I PI3K inhibitors.

Many growth factors, such as EGF and PDGF can stimulate cell chemotaxis. These responses are described as being inhibited by PI3K inhibitors in many studies. Use of fluorescent intracellular reporters have shown that PtdIns(3,4,5)P_3_ is concentrated at the leading edge of cells chemotaxing towards these ligands and that local generation or application of PtdIns(3,4,5)P_3_ may drive cell protusion. Current models suggest class I PI3Ks have an important role in sensing the direction of an external gradient of chemoattractant and translating that information into an intracellular gradient of PtdIns(3,4,5)P_3_ that shapes the direction of migration. This view has been challenged by work studying neutrophil chemotaxis, which suggests that class I PI3Ks have an important role in chemokinesis but not in direction-sensing and that any effects of PI3K inhibition on chemotaxis are indirect. In this context we found that EGF-stimulated both chemokinesis and chemotaxis of both MCF10a and MDA-MB 231 cells. Chemokinesis was inhibited by either pan-class I PI3K inhibitors or by PI3Kα-selective inhibitors or p110α-directed shRNAi specifically. However, chemotaxis was only inhibited in the sense that the cells chemotaxed for less distance and, because of that, the statistical clarity with which they could be demonstrated to have chemotaxed was reduced. Hence by recording the individual tracks of many cells in the presence of PI3K inhibitors or shRNAi it was clear that although the distance the cells moved under a given set of conditions was dramatically reduced that movement was still chemotactic. These results suggest that, like neutrophils responding to chemoattractants [93], [94], MCF10a cells and MDA-MB 231 cells require class I PI3K activity and PtdIns(3,4,5)P_3_ for chemokinesis but not the underlying ability to chemotax.

Our results, obtained through the use of inhibitors, shRNAi and expression of p110α-onco-mutants, show that PI3Kα has a unique and important role in regulation of chemokinesis. In MDA-MB 231 cells particularly it is clear that inhibition of other class I PI3Ks has little effect on chemokinesis and yet have effects on phosphorylation of PKB. These results suggest that PKB is unlikely to be an important target of PI3Kα in this pathway. This idea is supported by a number of other features of our results; that knock-in of a single onco-mutant allele of PKB had no effect on basal or EGF-stimulated chemokinesis and that suppression of p110α with shRNAi reduced EGF-stimulated chemokinesis strongly but reduced PKB phosphorylation weakly. This view apparently contrasts with a recent paper indicating PKB/mTOR may play a key role in suppressing an invasive, migratory phenotype in MCF10a cells [81]. However, that invasive phenotype was manifest in a 3D culture environment dependent on the coordinated action of many cell processes and the part played by chemokinesis in that is unclear. Our work does not reveal the identity of the targets of PtdIns(3,4,5)P_3_ signaling, that probably reside within the family of one hundred or so known, mammalian PtdIns(3,4,5)P_3_ -effectors, which are important for chemokinesis.

Heterologous expression or genetic knock-in of onco-mutant p110α constructs has been reported to enhance cell migration and/or invasiveness. Our results confirm these observations but reveal these effects are only clearly manifest in basal, unstimulated cells. Pan-class I PI3K inhibitors suppress EGF-stimulated chemokinesis but leave the onco-mutant induced increase in basal chemokinesis unchanged. A simple explanation of this result is that the chronic presence of the activated p110α allele leads to the induction of pro-chemokinetic processes that do not depend on PI3K activity acutely.

Loss of PTEN leads to a large decrease in the level of both basal and EGF-stimulated cell migration. This appears to correlate with cells adopting a highly spread, adhesive phenotype on tissue culture plastic that resists migration. This phenomenon has been described in other cell types and has been “reversed” by treatment with pan-class I PI3K inhibitors indicating the effect has resulted from increased PtdIns(3,4,5)P_3_. The implication of these observations is that loss of PTEN and the resulting increase in PtdIns(3,4,5)P_3_ does not phenocopy the result of expressing an onco-mutant p110α that also increases PtdIns(3,4,5)P_3_, because there is a multi-modal or dose-dependent effect of PtdIns(3,4,5)P_3_ on cell migration.

A broad conclusion of these experiments is that efficient inhibition of PI3Kα can have very different impacts on the scale of activation of PKB in different, but broadly similar, cancer cell types.

## Supporting Information

Figure S1 **Impact of class I PI3K inhibitors on cell growth and metabolism. Panel A.** PTEN^−/−^ MCF10a were cultured in full growth medium for 48 h in presence of DMSO (CT) or inhibitors (A66, 6 µM (left) or 2 µM (right); TGX221, 40 nM; IC87114, 1 µM; PIK75, 1 µM; “mix” A66, 6 µM+TGX221, 40 nM+IC87114, 1 µM; PI103, 1 µM). The growth and viability was measured using PrestoBlue and quantified by absorbance (570/595 nm). Data presented are means ± SE (n = 3, experiments). The p-values were obtained with a Dunnett test. **Panel B.** Same experiment was performed on MDA-MB468 cells. Data presented are means ± SE (n = 3, experiments). The p-values were obtained with a One-Way ANOVA followed by a Dunnett test.
(TIF)

Methods S1 **Growth and viability assay.**
(DOCX)
