# Supplementary figures and images for: Signaling via Class IA Phosphoinositide 3-Kinases (PI3K) in Human, Breast-Derived Cell Lines
Source: PLoS One. 2013 Oct 4;8(10):e75045. doi: 10.1371/journal.pone.0075045 (PMC3790768; doi:10.1371/journal.pone.0075045)

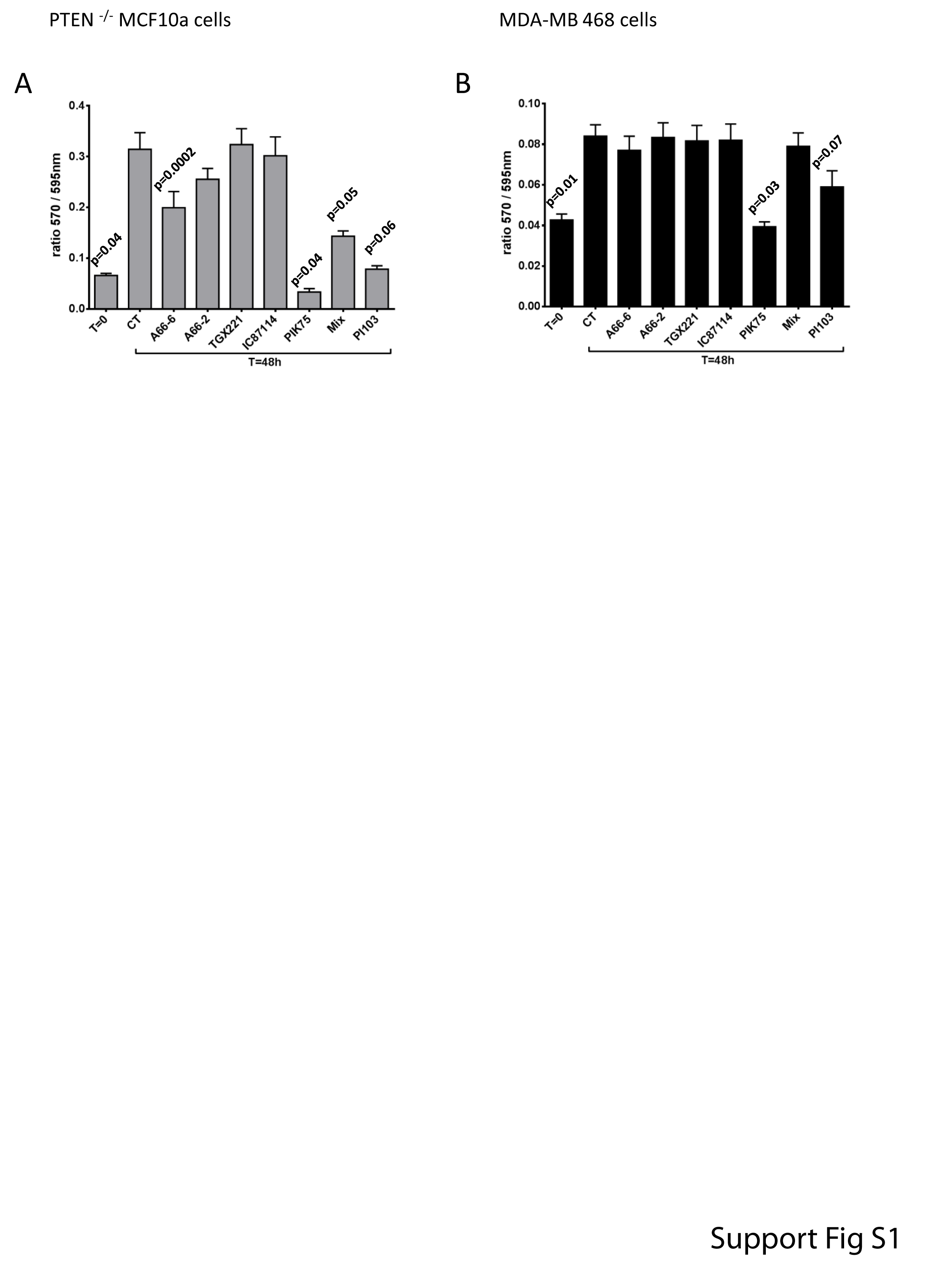

Supplement: Figure S1 — Impact of class I PI3K inhibitors on cell growth and metabolism. Panel A. PTEN−/− MCF10a were cultured in full growth medium for 48 h in presence of DMSO (CT) or inhibitors (A66, 6 µM (left) or 2 µM (right); TGX221, 40 nM; IC87114, 1 µM; PIK75, 1 µM; “mix” A66, 6 µM+TGX221, 40 nM+IC87114, 1 µM; PI103, 1 µM). The growth and viability was measured using PrestoBlue and quantified by absorbance (570/595 nm). Data presented are means ± SE (n = 3, experiments). The p-values were obtained with a Dunnett test. Panel B. Same experiment was performed on MDA-MB468 cells. Data presented are means ± SE (n = 3, experiments). The p-values were obtained with a One-Way ANOVA followed by a Dunnett test. (TIF) [file pone.0075045.s001.tif]
