# Supplementary material for: Signaling via Class IA Phosphoinositide 3-Kinases (PI3K) in Human, Breast-Derived Cell Lines
Source: PLoS One. 2013 Oct 4;8(10):e75045. doi: 10.1371/journal.pone.0075045 (PMC3790768; doi:10.1371/journal.pone.0075045)
Supplement: Methods S1 — Growth and viability assay. (DOCX) [file pone.0075045.s002.docx]

**Supporting Information Methods**

**Growth and viability assay.**

Cells were seeded in the morning in 96 well plates at 2x10^3^ cells/ 100ul/ well (3 wells per condition) in their growth medium and leave to attach. Once the cells attached 100ul of the medium containing drugs or vehicle was added to the appropriate wells. The control plates for T=0 were stopped at this point; the next ones were stopped 48h later: 10ul of PrestoBlue (Life technologies) was added in the well containing 90ul of medium + cells. After 2h incubation at 37’C, the absorbance was read at 570 nm and 595 nm. After subtraction of the background level (medium only + PrestoBlue), the ratio (570/595) has been calculated.
